# Effects of Foliar Boron Application on Physiological and Antioxidants Responses in Highbush Blueberry (*Vaccinium corymbosum* L.) Cultivars

**DOI:** 10.3390/plants13111553

**Published:** 2024-06-04

**Authors:** Marjorie Reyes-Díaz, Paz Cárcamo-Fincheira, Ricardo Tighe-Neira, Adriano Nunes-Nesi, Arnould Savouré, Claudio Inostroza-Blancheteau

**Affiliations:** 1 Laboratorio de Ecofisiología Molecular y Funcional de Plantas, Departamento de Ciencias Químicas y Recursos Naturales, Facultad de Ingeniería y Ciencias, Universidad de La Frontera, Temuco P.O. Box 54-D, Chile; marjorie.reyes@ufrontera.cl (M.R.-D.); paz.carcamo@ufrontera.cl (P.C.-F.); 2 Center of Plant, Soil Interaction and Natural Resources Biotechnology, Scientific and Technological Bioresource Nucleus (BIOREN-UFRO), Universidad de La Frontera, Temuco P.O. Box 54-D, Chile; 3 Laboratorio de Fisiología y Biotecnología Vegetal, Departamento de Ciencias Agropecuarias y Acuícolas, Facultad de Recursos Naturales, Universidad Católica de Temuco, Temuco P.O. Box 56-D, Chile; rtighe@uct.cl; 4 Núcleo de Investigación en Producción Alimentaria, Facultad de Recursos Naturales, Universidad Católica de Temuco, Temuco P.O. Box 56-D, Chile; 5 Departamento de Biologia Vegetal, Universidade Federal de Viçosa, Viçosa 36570-900, MG, Brazil; nunesnesi@ufv.br; 6 UPEC, CNRS, IRD, INRAE, Institute of Ecology and Environmental Sciences of Paris (iEES), Sorbonne Université, 75005 Paris, France; arnould.savoure@sorbonne-universite.fr

**Keywords:** ascorbic acid, cell wall, Solubor^®^, woody plants

## Abstract

Boron (B) is a micronutrient crucial for the growth, development, productivity, and quality of crops. However, in areas characterized by acid soil (pH_water_ < 5.0) and high rainfall, soil B concentration tends to decrease, leading to insufficient supply to crops. This study was aimed at determining the optimal rate of B fertilization to enhance *Vaccinium corymbosum* L. performance in acid conditions. One-year-old cultivars with contrasting Al resistance (Al-sensitive Star and Al-resistant Cargo) were used. Plants were conditioned in plastic pots containing 18 L of half-ionic-strength Hoagland solution (pH 4.5) for 2 weeks. Thereafter, the following B treatments were applied foliarly: control, without B application (distilled water), 200, 400, and 800 mg L^−1^ of B as Solubor^®^ for up to 72 h. Photosynthetic performance, root and shoot B levels, antioxidants, and oxidative stress were evaluated. Root and shoot B concentrations increased with the increasing B application, being higher in leaves than in roots of both cultivars. Net photosynthesis decreased at 800 mg L^−1^ B supply and effective quantum yield of PSII at 72 h in all B treatments. Lipid peroxidation increased in both cultivars at 800 mg L^−1^ B treatment. Antioxidant activity increased in all B treatments in both cultivars; while, at 400 and 800 mg L^−1^ B, total phenols increased in leaves of cultivar Star and decreased in cultivar Cargo. In conclusion, optimal B foliar application for highbush blueberry appears to be around 400 mg L^−1^ B. The appropriate B foliar application could help mitigate potential stress-induced problems in highbush blueberry cultivation. However, the optimal foliar B application should be confirmed in field experiments to help the farmers manage B nutrition.

## 1. Introduction

Boron (B) is essential for the growth, development, productivity, and quality of crops [1,2]. Its absorption by plants, primarily in the form of boric acid [B(OH)_3_], occurs through root membranes via passive diffusion [3,4]. Also, some plants possess efficient B transport systems activated under low or high B conditions; for example, under B deficiency, the boric acid channel NIP5;1 is activated in root cells of Arabidopsis for boric acid uptake, whereas xylem loading is performed by the borate transporter BOR1 [5]. 

The requirement of B for normal plant growth and development ranges from 10 to 75 mg kg^−1^ in dried leaf tissues for many crops. Boron requirements vary depending on the type of plants: in monocotyledons, leaf content ranges from 1 to 6 mg kg^−1^, while in dicotyledons, it ranges from 20 to 70 mg kg^−1^ [6]. Boron plays a pivotal role in the synthesis and the properties of the cell wall [7,8]. In addition, B is involved in the regulation of the synthesis of ascorbate and lignin, as well as the maintenance of the antioxidant system [6,9]. In this context, a recent study by [10] reported that the addition of boron could help to detoxify H^+^-toxicity by activating the antioxidant system, thereby reducing oxidative damage in the roots of trifoliate seedlings (*Poncirus trifoliate* (L) Raf.). In regions characterized by acid soil (pH_water_ < 5) and high rainfall, availability of B in soils is low, leading to plant deficiency manifested as abnormal growth in young plants and a rapid inhibition of root growth and elongation, subsequently affecting crop yield and quality [11]. Conversely, excessive B supply can result in decreased chlorophyll content; reduced photosynthetic capacity; and yield reduction in *Citrus* rootstock cultivars, *Cucurbita pepo*, *Cucumis sativus*, *Mentha arvensis*, and *Cymbopogon fexuosus* [8,12,13]. 

Cultivation of highbush blueberry (*Vaccinium corymbosum* L.), prevalent in southern Chile [14], occurs on volcanic ash-derived soils with high acidity and high aluminum concentrations that often coincide with low B availability [14,15,16]. In addition, elements such as manganese (Mn) increase in acid soils; although it is an essential micronutrient, its excess can produce toxic effects in plants [17,18]. In these acid soils, B is strongly adsorbed by the allophane, which is the most dominant mineral in the clay fraction [19,20], producing its deficiency. This problem is exacerbated by the high rainfall in autumn–winter, so foliar application of boron at the reproductive phenological stage (e.g., flowering) in spring–summer is very necessary for farmers to improve the fruit set, fruit production, and quality. To address these challenges, it is important to characterize the effects of variable B supply on highbush blueberry under acidic conditions as a theoretical basis for new agronomic strategies to mitigate these B-related problems. Therefore, this study was aimed at determining the optimal rate of foliar B application to enhance highbush blueberry performance under acidic conditions.

## 2. Results

### 2.1. B Concentration in Leaves and Roots of Highbush Blueberry Cultivars

**Figure 1:**
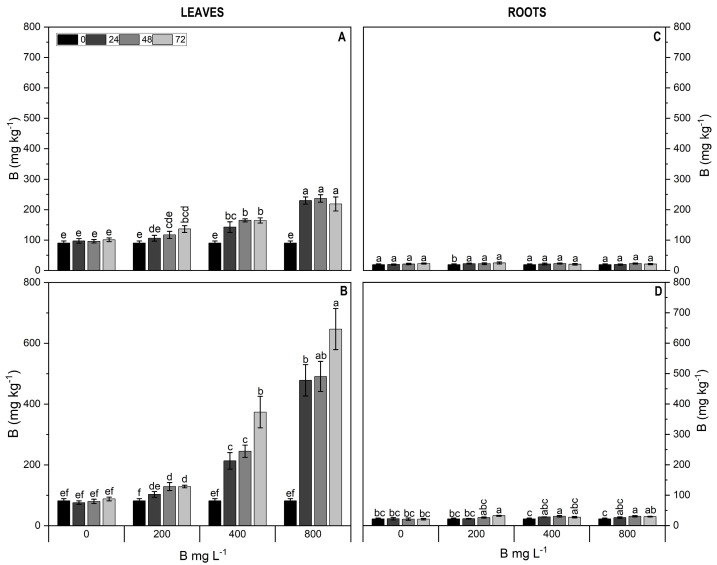
Boron concentration (mg kg^−1^) in leaves (**A**,**B**) and roots (**C**,**D**) of Al-resistant cultivar Cargo (**A**,**C**) and Al-sensitive cultivar Star (**B**,**D**) of *V. corymbosum* exposed to different rates of foliar B application (200, 400, and 800 mg L^−1^ Solubor^®^) for 0, 24, 48, and 72 h. The values are the averages of four independent biological replicates [±standard error (SE)]. Lowercase letters indicate significant differences (*p* ≤ 0.05) as influenced by the interaction treatment × time, according to the Tukey test.

To explore the uptake and translocation of B in plants under varying rates of B application, we performed analyses to determine the B concentration in roots and leaves of each cultivar (Figure 1). Our findings revealed a significant interaction between B doses and time (*p* < 0.05). In leaves, the Star cultivar exhibited the most substantial increase, reaching a 7.9-fold increase in the 800 mg L^−1^ treatment after 72 h, followed by a 4.6-fold increase at 400 mg L^−1^ after the same period following application, compared to the control (Figure 1B). Conversely, in Cargo leaves, these increments were more modest compared to Star (1.8-fold and 2.4-fold increases at 72 h for the 400 and 800 mg L^−1^ treatments, respectively) (Figure 1A). By contrast, both Star and Cargo roots showed no significant changes across different B doses and time (Figure 1C,D). 

### 2.2. Chlorophyll Fluorescence and Gas Exchange Parameters

**Figure 2:**
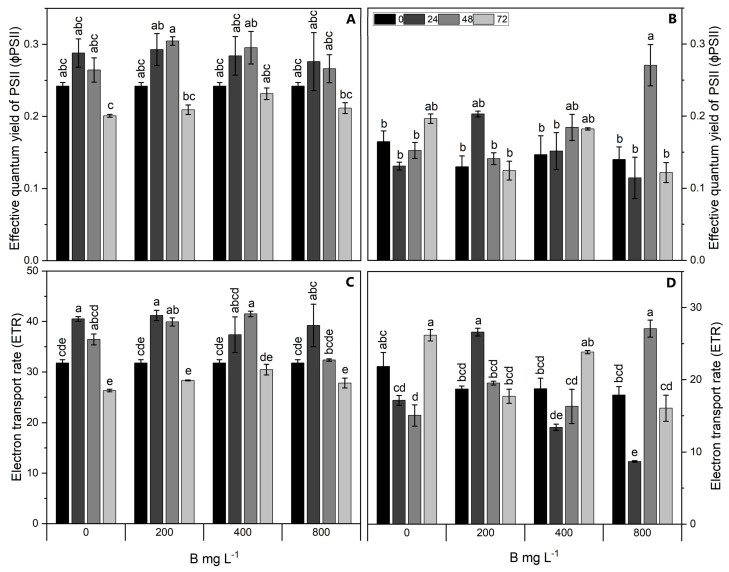
Fluorescence parameters of effective quantum yield of photosystem II (ΦPSII) (**A**,**B**) and electron transport rate (ETR) (**C**,**D**) of Al-resistant cultivar Cargo (**A**,**C**) and Al-sensitive cultivar Star (**B**,**D**) of *V. corymbosum* exposed to different rates of foliar B application (200, 400, and 800 mg L^−1^ Solubor^®^) for 0, 24, 48, and 72 h. The values are the averages of four independent biological replicates [±standard error (SE)]. Lowercase letters indicate significant differences (*p* ≤ 0.05) in the treatment × time interaction, according to the Tukey test.

**Figure 3:**
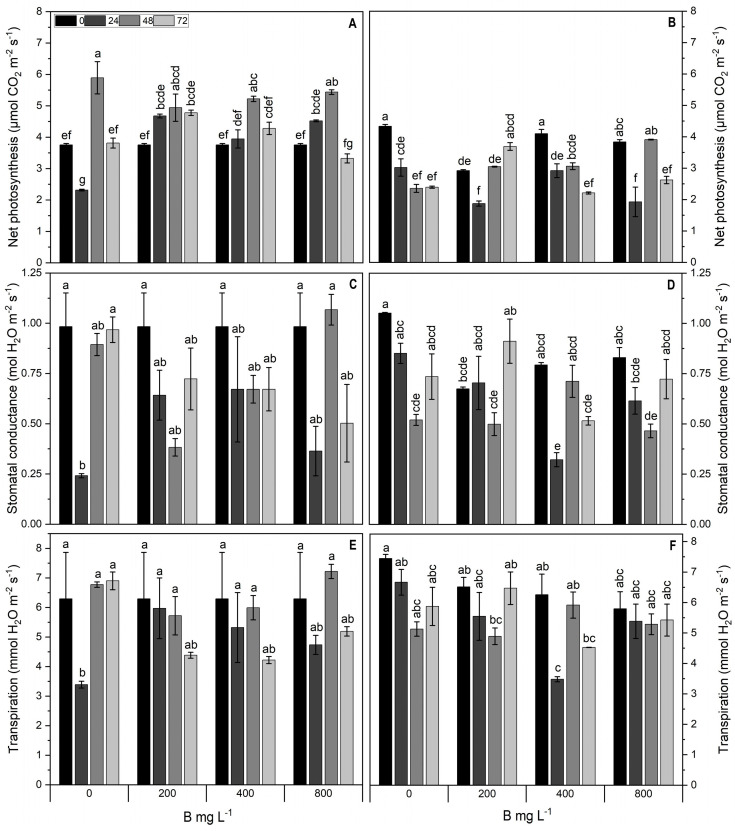
Photosynthetic parameters: net photosynthesis (Pn, µmol CO_2_ m^−2^ s^−1^) (**A**,**B**), stomatal conductance (gs, mol H_2_O m^−2^ s^−1^) (**C**,**D**), and transpiration (E, mmol H_2_O m^−2^ s^−1^) (**E**,**F**) of Al-resistant cultivar Cargo (**A**,**C**,**E**) and Al-sensitive cultivar Star (**B**,**D**,**F**) of *V. corymbosum* exposed to different rates of foliar B application (200, 400, and 800 mg L^−1^ Solubor^®^) for 0, 24, 48, and 72 h. The values are the averages of four independent biological replicates (±standard error [SE]). Lowercase letters indicate significant differences (*p* ≤ 0.05) in the treatment × time interaction, according to the Tukey test.

At 72 h in the Cargo cultivar, the effective quantum yield (ΦPSII) and electron transport rate (ETR) decreased in all treatments, except for 400 mg L^−1^ B, where no significant difference across time was recorded for both parameters (Figure 2A,C). Cultivar Star showed the highest increase in both parameters for the 800 mg L^−1^ B treatment at 48 h compared to the control (Figure 2B,D). A significant interaction between the B application rates and time (*p* < 0.05) was observed for the net photosynthesis (Pn) in both Star and Cargo. Cultivar Cargo showed an increase of up to 45% with an increase in the B dose over time compared to the control, except for 800 mg L^−1^ B at 72 h, where a decrease (13%) was observed (Figure 3A). In comparison, cultivar Star showed decreased Pn in all the treatments over time, except for 200 mg L^−1^ B at 72 h, where an increase of approximately 35% was observed (Figure 3B). In both cultivars, stomatal conductance (gs) exhibited fluctuations across treatments and times (Figure 3C,D). Transpiration (E) in both cultivars did not show any significant changes across treatments (Figure 3E,F). 

### 2.3. Photosynthetic Pigments

In Cargo, chlorophyll a, b, and total chlorophyll (a + b) in all treatments remained relatively stable across various B dose × time combinations; nonetheless, chlorophyll a and b were increased in the 200 mg L^−1^ B treatment compared to the control after 48 h (Table 1). In cultivar Star, chlorophyll a, a/b, and total chlorophyll (a + b) remained relatively stable in all treatments (Table 2). 

### 2.4. Assessment of Lipid Peroxidation and Antioxidant Activity 

**Figure 4:**
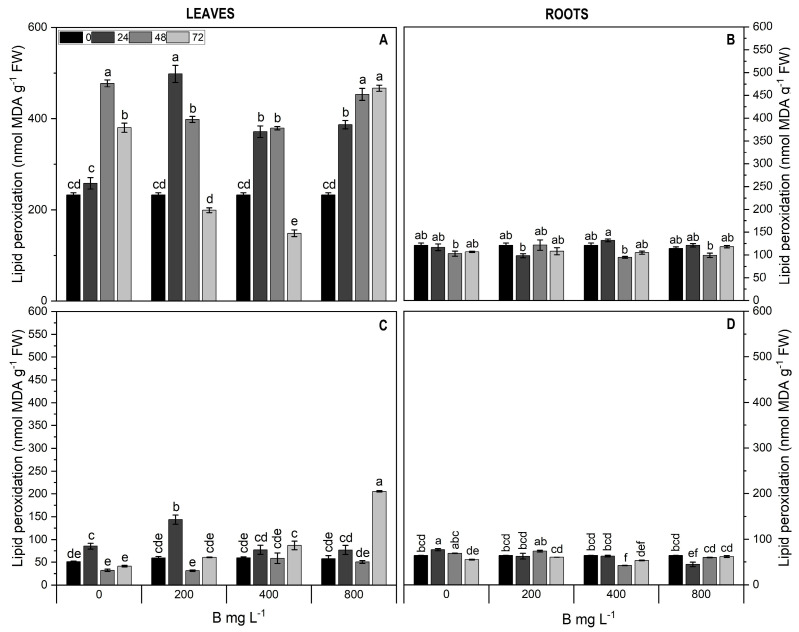
Lipid peroxidation in leaves (**A**,**C**) and roots (**B**,**D**) of Cargo (**A**,**B**) and Star (**C**,**D**) cultivars of *V. corymbosum* exposed to different doses of B (200, 400, and 800 mg L^−1^ Solubor^®^) for 0, 24, 48, and 72 h. The values are the averages of four independent biological replicates (±standard error [SE]). Lowercase letters indicate significant differences (*p* ≤ 0.05) in the treatment × time interaction, according to the Tukey test.

In Cargo leaves, lipid peroxidation increased progressively over time in the 800 mg L^−1^ B treatment, while noticeable decreases were observed at 72 h for the 200 and 400 mg L^−1^ B treatments (Figure 4). On the contrary, leaves of the Star cultivar did not show any significant differences in lipid peroxidation, except with 800 mg L^−1^ B at 72 h (Figure 4). In the roots of both cultivars, lipid peroxidation levels remained stable over treatments and time (Figure 4).

**Figure 5:**
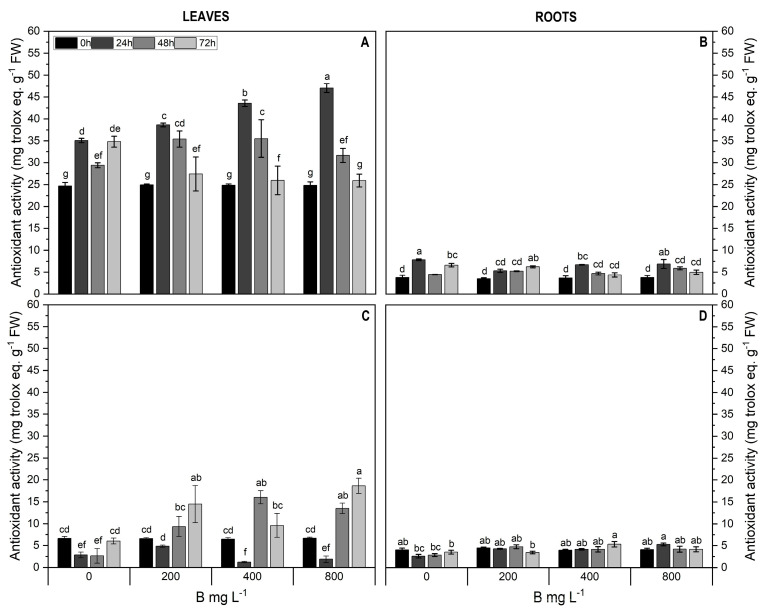
Total antioxidants (μg Trolox equivalents g^−1^ FW) in leaves (**A**,**C**) and roots (**B**,**D**) of Cargo (A,**B**) and Star (**C**,**D**) cultivars of *V. corymbosum* exposed to different doses of B (200, 400, and 800 mg L^−1^ Solubor^®^) for 0, 24, 48, and 72 h. The values are the averages of four independent biological replicates (±standard error [SE]). Lowercase letters indicate significant differences (*p* ≤ 0.05) in the treatment × time interaction, according to the Tukey test.

To elucidate the non-enzymatic antioxidant mechanism, we analyzed the contents of total antioxidants, total phenols, and total flavonoids. In Cargo leaves, total antioxidants doubled with 400 mg L^−1^ B treatment after 24 h compared to the control (Figure 5A). In Star leaves, total antioxidant levels were lower than those in Cargo, but increased up to 2.2-fold across treatments compared to the control (Figure 5C). Lower levels of total antioxidants were observed in roots of both cultivars compared to leaves, with a significant increase in roots of Cargo (1.9-fold) for the B treatments (Figure 5B,D).

**Figure 6:**
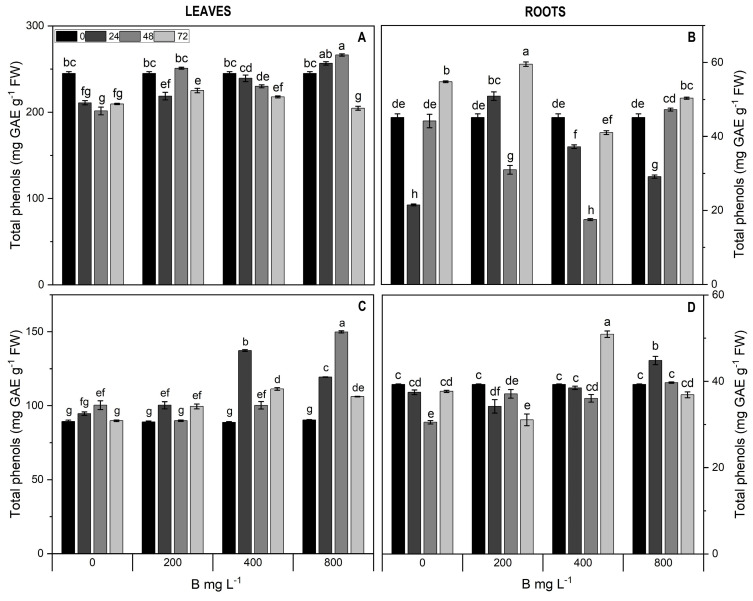
Total phenols in leaves (**A**,**C**) and roots (**B**,**D**) of Cargo (**A**,**B**) and Star (**C**,**D**) cultivars of *V. corymbosum* exposed to different doses of B (200, 400, and 800 mg L^−1^ Solubor^®^) for 0, 24, 48, and 72 h. The values are the averages of four independent biological replicates (±standard error [SE]). Lowercase letters indicate significant differences (*p* ≤ 0.05) in the treatment × time interaction, according to the Tukey test.

Total phenols showed a significant interaction between B doses and time (*p* < 0.01). In Cargo leaves, total phenols decreased by 16% with 800 mg L^−1^ B at 72 h, whereas in the treatment rates and durations, total phenols did not vary significantly compared to the control (Figure 6A). In cultivar Star leaves, total phenols increased only in the treatment with 400 mg L^−1^ B at 24 h (1.6-fold) and 800 mg L^−1^ B at 48 h (1.7-fold) (Figure 6C). In Cargo roots, total phenols showed a significant treatment-dependent decrease at 24 and 48 h, followed by recovery (Figure 6B). By contrast, in roots of Star, a significant increase (1.6-fold) was observed at 400 mg L^−1^ B after 72 h (Figure 6D). 

**Figure 7:**
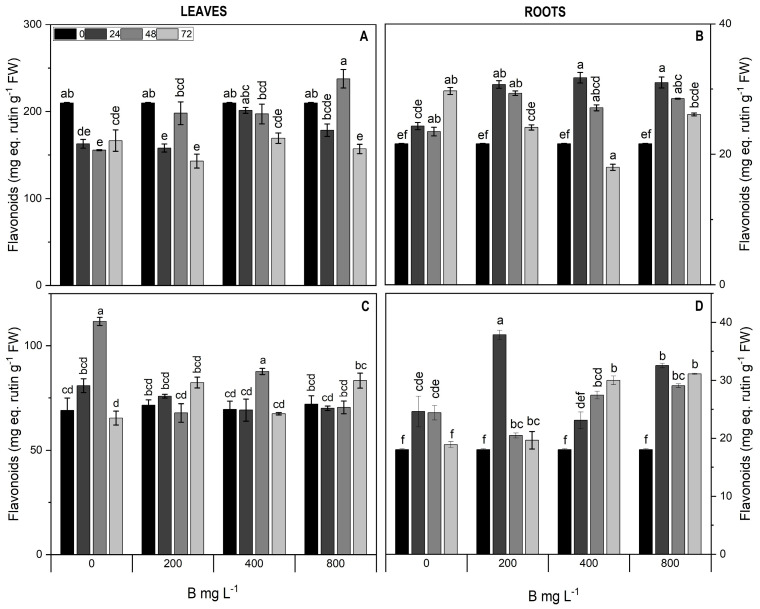
Total flavonoids in leaves (**A**,**C**) and roots (**B**,**D**) of Cargo (**A**,**B**) and Star (**C**,**D**) cultivars of *V. corymbosum* exposed to different doses of B (200, 400, and 800 mg L^−1^ Solubor^®^) for 0, 24, 48, and 72 h. The values are the averages of four independent biological replicates (±standard error [SE]). Lowercase letters indicate significant differences (*p* ≤ 0.05) in the treatment × time interaction, according to the Tukey test.

Total flavonoids in cultivar Cargo leaves decreased (up to 24.9%) across all treatments at 72 h compared to the control (Figure 7A), whereas in Star leaves, a slight increase was observed in all B treatments at 48 and 72 h (Figure 7C). In roots of both cultivars, total flavonoids increased (~31.9%) in all treatments compared to the control at 24 and 48 h, excepting in Star with 400 mg L^−1^ B at 24 h (Figure 7B,D).

**Figure 8:**
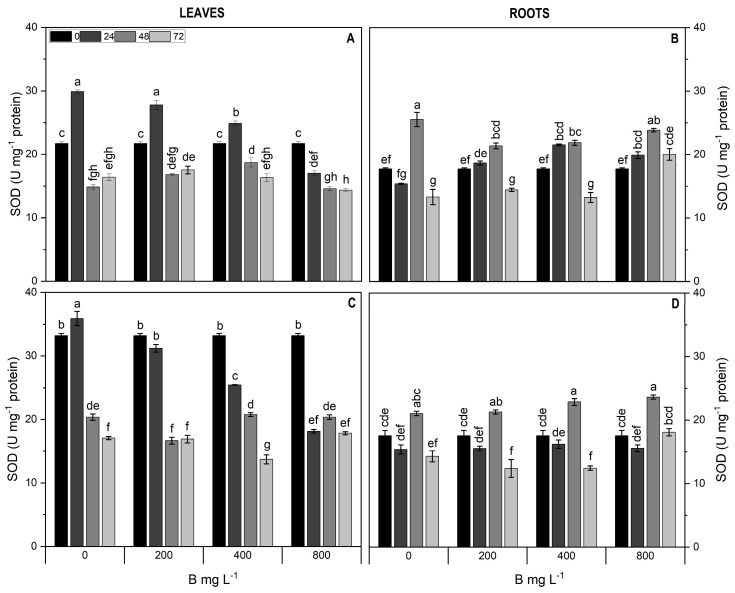
Superoxide dismutase activity in leaves (**A**,**C**) and roots (**B**,**D**) of Cargo (**A**,**B**) and Star (**C**,**D**) cultivars of *V. corymbosum* exposed to different doses of B (200, 400, and 800 mg L^−1^ Solubor^®^) for 0, 24, 48, and 72 h. The values are the averages of four independent biological replicates (±standard error [SE]). Lowercase letters indicate significant differences (*p* ≤ 0.05) in the treatment × time interaction, according to the Tukey test.

The SOD activity in the leaves of both cultivars increased across all treatments after 24 h, subsequently decreasing by up to 2.5-fold compared to the control. Similarly, in the roots of Cargo, a decrease (23.5%) in SOD activity was also found in the 200 and 400 mg L^−1^ B treatments at 48 h (Figure 8).

## 3. Discussion

The availability of B, in the form of boric acid (H_3_BO_3_) or borate anion [B(OH)_4_^−^], is directly related to the soil pH. At low pH, the predominant form is H_3_BO_3_, whereas at higher pH, the borate anion predominates [1,21]. In agriculture, the application of B fertilizer via foliar spraying has been recognized as a supplement to soil application to meet the plant B requirements [22]. Moreover, foliar application is the standard practice to rapidly alleviate B deficiency in plants [23,24,25,26]. 

The results of the present study revealed a higher accumulation of B in leaves compared to roots with increasing rates of B application, suggesting that B was not translocated from leaves to roots. It is known that B is highly mobile in the soil [27]; however, in plants, the long-distance mobility of B (from roots to the leaves) through the phloem depends on its ability to complex with other metabolites [28]. For instance, plants in the Oleaceae and Rosaceae families translocate B with large amounts of polyols in the phloem [29], while B shows low mobility in wheat (*Triticum aestivum*) and canola (*Brassica napus*), which translocate B complexed with sucrose in the phloem [30]. In Citrus (*Citrus* sp.), foliar-supplied B is transported from leaves to roots as a B–sucrose complex via phloem [26]. Our findings of higher B concentration in leaves of highbush blueberry corroborate the previous findings of [31], who reported a linear increase in B concentration with increasing rates of B application in beet (*Beta vulgaris*) and tomato (*Lycopersicon esculentum*). Similar results were observed in almond trees, where foliar application increased the B concentration in the tissues of this woody species [32,33,34]. These studies suggest that the boron application absorbed by leaves is likely transported as a B–sorbitol complex. Moreover, boric acid uptake occurs due to the high permeability and passive transport across plant membranes, whereas the responses of tolerant plant species to deficiency and toxicity nutrients may involve active transport [6,35]. 

Both B toxicity and deficiency present similar symptoms in plants, including a decrease in photosynthesis, and efficiency of photosystem II, low transpiration rate and stomatal conductance, alterations in the activity of antioxidative enzymes, and increased lipid peroxidation [36,37,38]. On the other hand, optimal B supply exerted a positive effect on gas exchange parameters such as the net CO_2_ assimilation rate and stomatal conductance in almond (*Prunus dulcis*) [39]. Similarly, in *Zea mays* plants, B application considerably improved growth, photosynthetic capacity, tissue B concentration, as well as the antioxidant defense system [40]. However, our results demonstrate alterations in the Pn and efficiency of the photosystem II under a low and high supply of B in both cultivars of *V. corymbosum*, possibly indicating symptoms of deficiency and/or toxicity. 

SOD activity plays a crucial role in plants under stress conditions, serving as an indicator of oxidative stress [13]. In boron-toxicity-tolerant plants like *Carthamus tinctorius* (safflower), low SOD activity was observed at high B concentrations [41], which is similar to the findings of this study. A similar behavior was observed in woody citrange orange species, when excess B resulted in decreased SOD activity and reduced activity of other antioxidant enzymes [42,43]. 

Generation of reactive oxygen species under B deficiency and toxicity leads to lipid peroxidation [43,44]. In our study, lipid peroxidation increased in both cultivars subjected to the 800 mg L^−1^ B application rate, consistent with findings reported in *Malus domestica*, *Solanum lycopersicum*, *Vitis vinifera*, *Mentha arvensis*, and apple rootstock under B toxicity [45,46,47,48]. On the other hand, increases in the concentration of phenolic compounds have been associated with boron deficiency due to the formation of the B-sugar cis-diol complex that regulates the accumulation of phenols [47]. In addition, higher total phenol content results from an increase in the activity of the enzyme phenylalanine ammonia lyase under B deficiency. Our results showed a peak in total phenol content in leaves of cultivar Star treated with 400 mg^−1^ B.

## 4. Materials and Methods

### 4.1. Plant Material and Growth Conditions

Two commercial cultivars of highbush blueberry (*Vaccinium corymbosum* L.) with different characteristics were used in this study: the Al-sensitive Star (USOOPP10675P) and the Al-resistant Cargo (US 2013023926OP1). These cultivars differ in aluminum resistance, genetic backgrounds, ecological adaptations, susceptibility to diseases, and soil and environmental requirements [48,49,50,51]. One-year-old plants (uniform size and foliar area with 40 cm in height) were conditioned in plastic pots containing 18 L of half-strength Hoagland solution [52] for 2 weeks. The pH was adjusted daily to 4.5. The composition of the nutrient solution was 3.0 mM KNO_3_, 2.0 mM Ca (NO_3_)_2_, 1.0 mM MgSO_4_, 0.1 mM KH_2_PO_4_, 1.0 mM NH_4_NO_3_, 20 µM Fe-EDTA, 25 µM H_3_BO_3_, 10 µM MnSO_4_, 0.4 µM CuSO_4_, 2.0 µM ZnSO_4_, and 0.07 µM (NH_4_)_6_Mo_7_O_24_. The growth chamber conditions were 16/8 h light/dark photoperiod, 22 ± 2 °C temperature, 70% relative air humidity, and light intensity around 300 μmol photons m^−2^ s^−1^. Foliar B application and treatments were control (distilled water) and 200, 400, and 800 mg L^−1^ of B as SOLUBOR^®^. The dose coverage of SOLUBOR^®^ solution was 10 mL per plant calculated as the total foliar area of the plant. Physiological parameters were immediately evaluated after B application (0 h) and at 24, 48, and 72 h after B application; samples of fully expanded leaves and roots were harvested in the morning between 08:00 and 10:00 h, rinsed extensively with distilled water, snap-frozen in liquid nitrogen, and stored at −80 °C until analysis.

### 4.2. Determination of B Concentration

Boron concentration was analyzed as described by [53], whereby shoot and root samples were dried at 70° C in a forced-air oven for 72 h. Then, 1.0 g of dried tissues was ground and dry-ashed in a muffle furnace at 500 °C for 8 h; the ash was then dissolved in 2 M HCl. The concentration of B was determined using a multi-element atomic absorption spectrophotometer (EAA, Model 969, Unicam, Cambridge, UK).

### 4.3. Chlorophyll Fluorescence and Gas Exchange Analyses 

Chlorophyll fluorescence was measured in light-adapted conditions, where the maximum quantum yield [Fv’/Fm’ = (Fm’ − 0’)/Fm’] was calculated according to [54], while the effective quantum yield of photosystem II [ΦPSII = (Fm’ − Fs)/Fm’)] and electron transport rate [ETR = ΦPSII × α × β × PPFD) were calculated according to [55]. Photosynthesis-related parameters were determined in fully expanded leaves as described by [56]. The parameters were net photosynthetic rate (Pn), stomatal conductance (gs), and transpiration (E); the measurements were performed early in the morning using a portable infrared gas analyzer (Licor LI6400, Lincoln, NE, USA) equipped with a measurement cuvette with its own light source (300 µmol photons m^−2^ s^−1^), with control of temperature (20 °C) and CO_2_ (400 mL L^−1^). 

### 4.4. Determination of Photosynthetic Pigment Contents 

Leaf samples (30 mg) were subjected to methanol extraction according to [57]. The photosynthetic pigments were determined as described by [58], using a microplate spectrophotometer (EPOCH, BioTek Instruments, Inc., Winooski, VT, USA) and measuring absorbances at 653 (chlorophyll a), 666 (chlorophyll b), and 470 nm (carotenoids) on a spectrophotometer (Genesys 10UV, Thermo Spectronic, Madison, WI, USA).

### 4.5. Lipid Peroxidation Assay 

The lipid peroxidation of the plasma membrane in leaves and roots of highbush blueberry was determined by the modified method described by [59], using thiobarbituric acid reacting substances (TBARS). Approximately 150 mg of ground fresh material was used for analysis. Absorbance was measured at 440, 530, and 660 nm by a spectrophotometer (Genesys 10UV, Thermo Spectronic, Madison, WI, USA).

### 4.6. Antioxidants Determination 

The antioxidant activity (AA) in roots and leaves was determined based on the method described by [60], using the 2.2-diphenyl-1-picrylhydrazyl (DPPH) free radical scavenging assay. Fresh root and leaf samples were ground in liquid nitrogen and soaked in 1 mL of 80:20 (*v*/*v*) methanol:water. The absorbance was measured at 515 nm by a spectrophotometer (Genesys 10UV, Thermo Spectronic, Madison, WI, USA), using Trolox as the standard. The values were expressed in μg Trolox equivalents g^−1^ fresh weight (FW). 

### 4.7. Phenol and Flavonoid Assays 

The total phenols (TPs) were determined in roots and leaves by the Folin–Ciocalteu method, as described by [61]. Absorbance was measured at 765 nm by a spectrophotometer and expressed in chlorogenic acid equivalents (CAEs) g^−1^ FW.

The flavonoids were determined in roots and leaves by the method described by [62]. Absorbance was measured at 510 nm by a spectrophotometer (Genesys 10UV, Thermo Spectronic, Madison, WI, USA) and expressed as mg of rutin equivalents per gram of fresh weight (mg rutin equivalent g^−1^ FW).

### 4.8. Superoxide Dismutase Activity

The SOD activity was determined according to [56], through the photochemical reduction of nitroblue tetrazolium (NBT). The reaction mixture contained 640 μL of 0.1 M potassium phosphate buffer (pH 7.0), 10 μL of 10 mM ethylenediaminete- traacetic acid (EDTA), 50 μL of 260 mM methionine, 80 μL of 4.2 mM NBT, 170 μL of 130 μM riboflavin, and 50 μL of supernatant. The reaction tubes were illuminated for 15 min, and the absorbance of the samples was measured at 560 nm in a microplate spectrophotometer (EPOCH, Bioteck Instruments, Inc., headquartered in Winooski, VT, USA). Non-illuminated and illuminated reaction mixtures without the supernatant were used as controls. One SOD unit was defined as the amount of enzyme corresponding to 50% inhibition of the NBT reduction [63]. The SOD activity was calculated on a protein basis (proteins measured according to the [64] method).

### 4.9. Experimental Design and Statistical Analyses

The experiment was performed in a randomized complete block design with two cultivars, four B treatments, three replicates, and three measurement times for the physiological and biochemical analyses. When the data passed the Kolmogorov–Smirnov test for the normality and homogeneity of variances, we performed two-way ANOVA (where the factors were B doses and sampling times) and used Tukey’s test (*p* ≤ 0.05) for mean comparisons. All analyses were performed using XLSTAT-LifeScience v.2022.

## 5. Conclusions

The foliar application experiment with B under greenhouse conditions and using a hydroponic solution revealed that 400 mg L^−1^ of B is necessary for the optimal physiological performance of highbush blueberry plants. However, the optimal B supply rates for highbush blueberry should be confirmed in field experiments to assist farmers in managing B nutrition effectively, because boron nutrition depends on various factors, including the soil pH, B status of plants, plant age, and more. Our study demonstrated that at a dose of 400 mg L^−1^ B, the B concentration significantly increased in both evaluated cultivars. Below this dose, highbush blueberry plants may experience deficiency, whereas higher doses may lead to B toxicity. Finally, this information demonstrates the importance of determining the optimal B dose to improve plant performance. By establishing the appropriate B application rates, growers can effectively manage B availability and supply, thereby mitigating potential stress-induced problems in blueberry cultivation. 

## Figures and Tables

**Table 1:** Photosynthetic pigments in leaves of *V. corymbosum* cultivar Cargo.

Time (h)–B Rate (mg L^−1^)	Chl a	Chl b	Chl a/b	Carotenoids
0-0	0.49 ± 0.046 b	0.21 ± 0.018 ab	2.34 ± 0.035 a	0.82 ± 0.011 abc
0-200	0.49 ± 0.046 b	0.21 ± 0.018 ab	2.34 ± 0.035 a	0.82 ± 0.011 abc
0-400	0.49 ± 0.046 b	0.21 ± 0.018 ab	2.34 ± 0.035 a	0.82 ± 0.011 abc
0-800	0.49 ± 0.046 b	0.21 ± 0.018 ab	2.34 ± 0.035 a	0.82 ± 0.011 abc
24-0	0.45 ± 0.066 b	0.21 ± 0.017 ab	2.40 ± 0.036 a	0.70 ± 0.026 c
24-200	0.45 ± 0.021 b	0.20 ± 0.009 b	2.24 ± 0.115 a	0.80 ± 0.046 abc
24-400	0.40 ± 0.037 b	0.17 ± 0.012 b	2.29 ± 0.088 a	0.72 ± 0.068 bc
24-800	0.54 ± 0.061 b	0.22 ± 0.017 ab	2.37 ± 0.096 a	0.98 ± 0.087 abc
48-0	0.41 ± 0.038 b	0.18 ± 0.012 b	2.17 ± 0.059 a	0.78 ± 0.065 abc
48-200	0.77 ± 0.018 a	0.29 ± 0.006 a	2.52 ± 0.846 a	1.09 ± 0.151 a
48-400	0.57 ± 0.016 ab	0.25 ± 0.004 ab	2.30 ± 0.094 a	1.00 ± 0.011 abc
48-800	0.59 ± 0.048 ab	0.24 ± 0.019 ab	2.40 ± 0.028 a	1.07 ± 0.112 ab
72-0	0.43 ± 0.018 b	0.18 ± 0.008 b	2.31 ± 0.108 a	0.74 ± 0.030 bc
72-200	0.53 ± 0.039 b	0.22 ± 0.013 ab	2.34 ± 0.032 a	0.95 ± 0.072 abc
72-400	0.57 ± 0.016 ab	0.25 ± 0.037 ab	2.31 ± 0.217 a	0.91 ± 0.087 abc
72-800	0.55 ± 0.025 b	0.21 ± 0.011 ab	2.35 ± 0.111 a	0.93 ± 0.030 abc ^1^

^1^ Different lowercase letters in the columns indicate significant differences by Tukey’s test at 5% significance. Values represent the mean ± SE.

**Table 2:** Photosynthetic pigments in leaves of *V. corymbosum* cultivar Star.

Time (h)–B Rate (mg L^−1^)	Chl a	Chl b	Chl a/b	Carotenoids
0-0	0.61 ± 0.050 ab	0.27 ± 0.021 abc	2.27 ± 0.011 a	1.08 ± 0.102 abc
0-200	0.59 ± 0.042 ab	0.26 ± 0.01 abcde	2.28 ± 0.003 a	1.16 ± 0.027 a
0-400	0.61 ± 0.018 ab	0.28 ± 0.02 abc	2.29 ± 0.001 a	1.11 ± 0.040 ab
0-800	0.61 ± 0.030 ab	0.27 ± 0.005 abc	2.27 ± 0.003 a	1.02 ± 0.008 abcd
24-0	0.45 ± 0.013 b	0.21 ± 0.005 cde	2.22 ± 0.055 ab	0.79 ± 0.038 cd
24-200	0.53 ± 0.010 ab	0.24 ± 0.011 abcde	2.18 ± 0.064 abc	0.92 ± 0.022 abcd
24-400	0.60 ± 0.057 ab	0.29 ± 0.001 ab	2.22 ± 0.046 ab	1.10 ± 0.100 abc
24-800	0.44 ± 0.045 b	0.19 ± 0.005 e	2.11 ± 0.029 abc	0.75 ± 0.019 d
48-0	0.59 ± 0.018 ab	0.25 ± 0.005 abcde	2.28 ± 0.068 a	0.95 ± 0.053 abcd
48-200	0.64 ± 0.005 a	0.26 ± 0.031 abcd	2.17 ± 0.010 abc	0.88 ± 0.085 abcd
48-400	0.54 ± 0.027 ab	0.19 ± 0.009 de	2.21 ± 0.058 abc	0.95 ± 0.07 abcd
48-800	0.51 ± 0.046 ab	0.30 ± 0.010 a	1.89 ± 0.104 c	0.85 ± 0.08 abcd
72-0	0.49 ± 0.010 ab	0.21 ± 0.002 cde	2.28 ± 0.072 a	0.84 ± 0.001 bcd
72-200	0.49 ± 0.033 ab	0.23 ± 0.010 abcde	2.07 ± 0.064 abc	0.85 ± 0.074 abcd
72-400	0.59 ± 0.041 ab	0.30 ± 0.016 a	1.93 ± 0.130 bc	1.02 ± 0.07 abcd
72-800	0.46 ± 0.005 b	0.22 ± 0.005 bcde	2.06 ± 0.07 abc	0.82 ± 0.02 bcd ^1^

^1^ Different lowercase letters in the columns indicate significant differences by Tukey’s test at 5% significance. Values represent the mean ± SE.

## Data Availability

All data supporting the findings of this study are available within the paper.
